# The Chinese translation and cross-cultural adaptation of the adult Grit Psychological Resources Scale

**DOI:** 10.3389/fpsyg.2026.1721845

**Published:** 2026-04-14

**Authors:** Wenlong Fan, Dongyang Wang, Fengting Nie, Ling Zhan, Shanghui Sun, Lijuan Dong

**Affiliations:** 1 Department of Nursing, Zhongshan Hospital of Traditional Chinese Medicine, Guangzhou, Guangdong, China; 2 Department of Nursing, The Third People’s Hospital of Henan Province, Zhengzhou, Henan, China

**Keywords:** Chinese translation, cross-cultural adaptation, grit, psychological resources, resilience

## Abstract

**Objective:**

The purpose of this study is to translate the Grit Psychological Resources Scale (GPRS) for adults into Chinese and to evaluate the reliability and validity of the translated version in a Chinese population.

**Methods:**

In this study, the scale was subjected to transcription, back-translation, and cross-cultural adaptation using Brislin’s dual translation-back translation method for pre-testing, to create an initial Chinese version of the Grit Psychological Resources Scale for adults. Convenience sampling was utilized to select 576 study participants from Shandong and Henan, China, who met the inclusion and exclusion criteria, to assess the scale’s reliability and validity. The internal consistency and test–retest reliability of the Chinese version of the GPRS were analyzed. Factor analysis was conducted to examine the factor structure of the scale.

**Results:**

The Chinese version of the Grit Psychological Resources Scale (GPRS) comprises 4 dimensions and 20 items. The overall Cronbach’s alpha coefficient for the questionnaire is 0.956. The split-half reliability of the scale is 0.736, and the test–retest reliability is 0.763. Exploratory factor analysis yielded a Kaiser-Meyer-Olkin (KMO) value of 0.949, and the Bartlett’s test of sphericity approximated a chi-square value of 5199.850 (*p* < 0.001). Principal Axis Factoring (PAF) was used to extract factors with eigenvalues greater than 1 as the main components, revealing a total of 4 principal components that account for 79.297% of the total variance explained. In confirmatory factor analysis, the model fit results were X2/df = 1.473, GFI = 0.92, AGFI = 0.90, NFI = 0.96, IFI = 0.99, TLI = 0.98, CFI = 0.99, RMSEA = 0.04. All model fit indices are within the acceptable range.

**Conclusion:**

The Chinese version of the Grit Psychological Resources Scale demonstrates good psychometric properties, making it suitable for assessing the psychological resources of grit in middle-aged and older Chinese adults. Future studies are needed to validate the scale in younger populations.

## 1 Introduction

Psychological resources typically refer to personal characteristics such as self-efficacy, optimism, Grit, and communication and problem-solving skills (Schimschal et al., 2023; Taylor et al., 2000). These characteristics serve as protective capital that can be used to buffer stress and maintain mental and physical health. In the context of grit, psychological resources can enhance people’s ability to cope with stressful situations and find various ways to stick to their passions, rather than giving up or changing direction like less gritty individuals. Therefore, assessing an individual’s resilience psychological resources is a crucial step in personal development.

Psychological resources can be applied to businesses and are at the core of human resources. In 1989, Hobfoll proposed the Conservation of Resources theory, which centers around resources and provides theoretical explanations for organizational psychology and behavior in the workplace. The definition and classification of individual resources have been proposed by Hobfoll to include four types of resources: material, conditional, personal characteristics, and energy. In recent years, psychological scientists have recognized the importance of exploring non-cognitive predictive factors for success in work, school, and other life domains (Datu, 2021). Psychological resources are gradually being applied to the public health sector. Both the fields of preventive medicine and positive psychology have set ambitious goals, which are not only to reduce disease risks but also to extend healthy life expectancy, improve quality of life, maintain good mental health resources and cognitive functions, and achieve healthcare savings.

Grit refers to “perseverance and passion for long-term goals,” characterized by maintaining focus in the face of setbacks. It is the driving force to achieve challenging ambitions (Lee et al., 2021). Research indicates that individuals with stronger willpower tend to perform better across various domains, including academic achievement (Halperin and Eldar Regev, 2021). In the past decade, grit has received increasing attention not only in empirical research but also in the public and educational sectors (Clark and Malecki, 2019). Numerous assessment scales have been developed, such as the Academic Persistence Scale (Clark and Malecki, 2019), the Sports Perseverance Scale (Guelmami et al., 2022), and others, which are closely related to and impact various aspects of life (Postigo et al., 2021).

A multitude of scales have been developed to assess adult grit, yet there is still no consensus on the definition or measurement of grit (Fekih-Romdhane et al., 2022). Grit was initially conceptualized as a multidimensional structure involving two facets: consistency of interests and perseverance of effort (Duckworth et al., 2007). The Grit Scale, developed and validated by Duckworth et al., was created to illustrate these dimensions (Gonzalez et al., 2020). The Short Grit has been validated in various countries and contexts, such as China (Li et al., 2018), South Korea (Kim and Lee, 2015), and Spain (Arco-Tirado et al., 2018). However, related research has pointed out that the scale is highly overlapping with other variables, which should reflect different constructs (i.e., conscientiousness, self-efficacy, self-control, and motivation). Postigo et al. (2021) developed and validated a new grit instrument, the Oviedo Grit Scale, in a Spanish-speaking population sample. Subsequently, Fekih-Romdhane et al. (2022) translated it into Arabic. However, these scales provide a more general assessment and do not specifically measure the level of gritty behavior. In fact, the current management of a large number of questionnaire items can have a negative impact on response rates and the accuracy of answers. Credé et al. (2017) conducted a meta-analysis and discovered that while grit, as a predictor of performance and success, has significant intuitive appeal as a focus of intervention, the current measures of grit do not seem to particularly predict success and performance. Additionally, it appears not to be markedly different from conscientiousness. They recommend that a re-evaluation of the Grit construct should be considered (Clark and Malecki, 2019).

Schimschal et al. (2022a) developed the GPRS (Grit Psychological Resource Scale) based on the grit psychological resource model to assess the level of psychological resources related to grit in adults. The model, referencing Duckworth’s previous work, identifies interest, purpose, practice, and hope as key assets for enhancing the quality of grit (Schimschal et al., 2022b). Currently, there is no research supporting the use of the GPRS for the Chinese population. Against this backdrop, considering the importance of this tool, the purpose of this study is to translate the scale into Chinese and to carry out cross-cultural adaptation and evaluation.

## 2 Methods

### 2.1 Study design

This study is a cross-sectional research conducted from May to August 2024, distributing an electronic survey link via Questionnaire Star to Chinese residents living in Shandong and Henan provinces, China. The researchers contacted the original author (Dr. Sarah E Schimschal) via email, explained the purpose and significance of this study, and obtained her authorization and consent to translate and culturally adapt the GPRS scale (Schimschal et al., 2022a). This study was conducted in accordance with the guidelines set forth in the Declaration of Helsinki. Approval number: 20240015.

### 2.2 Participants

All participants were informed and consented to take part in this study. A total of 576 subjects were included in the final analysis. The inclusion criteria were as follows: the subjects were Chinese adults, residing in Shandong and Henan provinces, China, aged 18 or above, and capable of reading and understanding the Chinese language. Subjects who did not meet the study’s inclusion criteria were excluded from participation. An electronic questionnaire was created using an online survey platform.^1^ The survey link included the Chinese version of the GPRS as well as other questionnaires assessing theoretically related outcomes and constructs. The link was distributed via email and various social media platforms (Wechat App). Participants were only allowed to respond to the questionnaire once. The survey provided contact information for the principal investigator for any inquiries. The study’s inclusion and exclusion criteria, plan and procedures, as well as the rights of the participants, were explained at the beginning of the survey. All participants were assured that their privacy would be respected and that no personal information would be disclosed to the public.

### 2.3 Materials

#### 2.3.1 The Grit Psychological Resources Scale (GPRS)

The scale consists of 20 items, divided into four subscales: Interest, Purpose, Practice, and Hope. Participants rate the items on a 7-point Likert scale, ranging from 1 (strongly disagree) to 7 (strongly agree). Higher scores indicate better psychological resources of grit.

#### 2.3.2 Criterion-related instruments

To evaluate the convergent validity in the external sense, the 10-item Connor-Davidson Resilience Scale (CD-RISC-10) was used as a criterion. The CD-RISC-10 is widely used to assess psychological resilience, which is theoretically highly correlated with grit. It uses a 5-point Likert scale, with higher scores indicating greater resilience. In this study, the Cronbach’s *α* for the CD-RISC-10 was 0.89.

### 2.4 Procedure

#### 2.4.1 Translation and cross-cultural adaptation of the Grit Psychological Resources Scale

In accordance with guidelines for cross-cultural adaptation, the translation process from English to Chinese is as follows. ① Primary translation: Two independent doctoral students majoring in Psychology, who are native speakers of Chinese, were tasked with translating the scale into Chinese. ② Synthesis of translations: A nursing doctoral graduate with overseas study experience was invited to compare the two translated versions, to propose revisions, reconcile discrepancies between the translations, and ensure that the translated items fully express the meaning of the original scale’s items, thereby forming a harmonized Chinese version of the translation. ③ Back-translation: A bilingual expert with proficient knowledge in psychology, fluent in both Chinese and English, performed a back-translation of the forward-translated version into English, without reference to the original version. ④ Expert panel consensus: A committee of six experts was purposefully selected, comprising two nursing management experts, two psychologists, one public health researcher, and one bilingual linguistics expert. The inclusion criteria for experts were holding a senior professional title or a PhD, and having at least 10 years of experience in their respective fields. The committee engaged in a comprehensive discussion regarding the semantic, conceptual, and habitual expressions of each item across the original scale, the harmonized translation, and the back-translated versions. To quantify content validity, experts rated each item on a 4-point Likert scale (1 = not relevant, 4 = highly relevant). The item-level content validity index (I-CVI) and scale-level content validity index (S-CVI) were calculated. They refined and integrated these aspects to finalize the back-translated integrated version across the original scale, the harmonized translation, and the back-translated versions. They refined and integrated these aspects to finalize the back-translated integrated version, which was then submitted to the original author for review and validation. In the event that the back-translated integrated version of the scale contains expressions that are not precise or do not align with the original scale’s items, the aforementioned translators and back-translators will engage in a recursive process of translation, back-translation, and communication with the original author to address these discrepancies. This iterative refinement will continue until a consensus is reached with the original author, culminating in the formation of the final Chinese inquiry version. ⑤ Pilot testing: A preliminary face-to-face test was conducted involving a convenience sample of 30 adult subjects recruited from a local hospital and community in Zhengzhou. While the main study utilized an online survey to achieve a larger sample size, this pilot phase was intentionally conducted in-person. The research team provided one-on-one on-site observation to specifically assess item comprehension, the clarity of wording, the time required to complete the survey, and overall acceptability. In the event of any inquiries during the completion process, standardized instructions were utilized to clarify and ensure uniform understanding. The average completion time was approximately 8 min, and participants reported that the items were easy to understand. In the event of any inquiries during the completion process, standardized instructions were utilized to clarify and ensure uniform understanding.

#### 2.4.2 Statistical analysis

Data entry and verification were accomplished utilizing EpiData software, ensuring the accuracy of the data through dual-entry checks. Subsequent data analysis was conducted employing IBM SPSS version 26.0 and Amos 24.0 software (IBM Corp., Armonk, NY, USA). The sample, comprising 576 participants, was divided into two datasets for Confirmatory Factor Analysis (CFA, nA = 288) and Exploratory Factor Analysis (EFA, nB = 288). We scrutinized the datasets for any missing data and outliers. Continuous variables were represented through means and standard deviations (SD), while categorical variables were expressed in terms of frequency (*n*) and percentage (%).

#### 2.4.3 Item analysis

Initially, we employed the critical ratio method and homogeneity testing for item analysis. The objective of item analysis is to eliminate items from the scale that do not meet specified criteria through statistical correlation tests. In this study, we primarily utilized the correlation coefficient test and the critical ratio test for item analysis. The correlation coefficient test involves determining the corrected item-total correlation, which calculates the correlation coefficient between the score of an individual item and the total score of the remaining items on the scale (i.e., total score excluding that specific item). If the significance level corresponding to a particular item’s correlation coefficient is less than 0.05, it is concluded that the item has a statistically significant test result, indicating good quality of the item, and thus, it should be retained. The critical ratio method primarily utilizes the independent samples T-test for comparative analysis. Specifically, the total scores of the scale are arranged in ascending order, and the samples scoring in the top 27% range are defined as Group 1, while those in the bottom 27% range are defined as Group 2. An independent samples T-test is conducted on these two groups of data. If the T-statistic for a particular item corresponds to a significance level less than 0.05, it indicates that the item possesses good quality and should be retained. Conversely, if the significance level is higher, the item should be considered for deletion.

#### 2.4.4 Validity analysis

We conducted the Kaiser-Meyer-Olkin (KMO) test and Bartlett’s test of sphericity to ensure the data’s suitability for factor analysis. To determine the number of factors, we utilized the Kaiser criterion (eigenvalues > 1.0), Cattell’s scree test (Ledesma et al., 2015), and Parallel Analysis, which is considered a more robust and defensible factor-retention strategy. To ascertain the underlying latent factor structure of the Grit Psychological Resources Scale (GPRS), we performed Principal Axis Factoring (PAF) rather than PAF, applying Varimax rotation. The acceptable threshold for item retention was defined as a primary factor loading of ≥ 0.50, with no cross-loadings ≥ 0.32 on any other factor. Subsequently, Confirmatory Factor Analysis (CFA) was conducted using AMOS software to evaluate the construct validity of the scale (Flora and Curran, 2004). Maximum Likelihood (ML) estimation was employed for the CFA. Given that the GPRS utilizes a 7-point Likert scale, the items were treated as continuous variables. Methodological literature supports that ordinal data with five or more categories can be robustly analyzed using ML estimation without significant parameter bias, especially when the data approximate a normal distribution. The model fit was determined using the following critical criteria: the Chi-square to degrees of freedom ratio (X2/df), the Comparative Fit Index (CFI > 0.9), the Chi-square fit statistic per degree of freedom (CMIN/DF < 5), the Goodness of Fit Index (GFI > 0.9), and the Root Mean Square Error of Approximation (RMSEA) (< 0.08).

#### 2.4.5 Reliability analysis

Reliability refers to the consistency or stability of measurement outcomes, that is, the degree to which a scale produces consistent results when the measurement is repeated. In this study, the overall reliability of the questionnaire was measured using Cronbach’s alpha coefficient, and the contribution of each item to the overall internal consistency reliability was assessed by examining the change in Cronbach’s alpha upon their deletion.

Should the deletion of a particular item result in a Cronbach’s alpha coefficient higher than that of the overall scale, it indicates that the presence of this item does not contribute to the overall internal consistency reliability. In fact, removing the item can enhance the composite Cronbach’s alpha value, thus warranting the item’s exclusion (Leppink and Pérez-Fuster, 2017).

To provide a more robust estimate of internal consistency, McDonald’s *ω* was also computed. For the assessment of test–retest reliability, a convenience subsample of 50 participants was invited to complete the GPRS again 2 weeks after their initial assessment.

## 3 Results

### 3.1 General information

A total of 576 individuals participated in this study, comprising 213 males (36.98%) and 363 females (63.02%). The sample was geographically concentrated in two provinces: Shandong (*n* = 240, 41.7%) and Henan (*n* = 336, 58.3%). The mean age of the participants was 50.53 years with a standard deviation of 5.06 years. The total score of the Chinese version of the GPRS among the participants was 102.45 ± 15.68 (range: 58–140). The mean scores for the four subscales were 24.52 ± 4.21 for Interest, 26.85 ± 3.95 for Purpose, 25.18 ± 4.12 for Practice, and 25.90 ± 4.08 for Hope.

The data for the 20 items were distributed across a range of 1 to 7, with average scores falling between 4 and 5, indicating a relatively uniform and stable distribution. Additional sociodemographic data are presented in Table 1.

**Table 1:** Sociodemographic characteristics.

Variable	*n* (%)
Age (years)	50.53 ± 5.06 (range: 35–68)
Gender	
Male	213 (36.98%)
Female	363 (63.02%)
Degree of education	
Primary school and below	57 (9.90%)
Junior high school	136 (23.61%)
Secondary vocational or high school	198 (34.38%)
College or bachelor’s degree and above	176 (30.56%)
Marital status	
Married	527
Divorce	43
Widowed	6

### 3.2 Item analysis

An assessment of the 20 items included in the GPRS was conducted, with results presented in Table 2.

**Table 2:** Project analysis results table.

Question items	Critical ratio value method	Corrected item-total correlation	Conclusion
	CR	R	
GPRS1: I have been seeking to learn new things	29.110^***^	0.802^***^	Reserve
GPRS2: I am usually very aware of my behavior	29.997^***^	0.791^***^	Reserve
GPRS3: I can work outside of my comfort zone	25.203^***^	0.773^***^	Reserve
GPRS4: I usually persist in studying	25.777^***^	0.765^***^	Reserve
GPRS5: I can tolerate uncertainty on the path forward	30.110^***^	0.796^***^	Reserve
GPRS6: I am committed to achieving my goals	20.234^***^	0.704^***^	Reserve
GPRS7: I pursue activities that are valuable to me	19.445^***^	0.718^***^	Reserve
GPRS8: My goals provide the direction of the have a purpose	19.061^***^	0.707^***^	Reserve
GPRS9: I am trying to learn from bad experiences	19.618^***^	0.695^***^	Reserve
GPRS10: When faced with difficulties, I can overcome negative emotions	21.894^***^	0.740^***^	Reserve
GPRS11: Generally speaking, I will keep trying until I figure out the problem	20.737^***^	0.716^***^	Reserve
GPRS12: I am good at dealing with challenges	20.139^***^	0.692^***^	Reserve
GPRS13: I usually pick myself up when I encounter difficulties	21.443^***^	0.712^***^	Reserve
GPRS14: I have the ability to achieve success	19.834^***^	0.690^***^	Reserve
GPRS15: I am very clear about the goal I want to achieve	22.169^***^	0.735^***^	Reserve
GPRS16: Continuous improvement is very important to me	20.104^***^	0.716^***^	Reserve
GPRS17: Feedback can help me make progress	22.177^***^	0.743^***^	Reserve
GPRS18: I can improve my abilities through hard work	23.396^***^	0.759^***^	Reserve
GPRS19: I have found that most problems can be solved	21.053^***^	0.735^***^	Reserve
GPRS20: I can focus on the overall situation	23.114^***^	0.742^***^	Reserve

*Indicates *p* < 0.05, ** indicates *p* < 0.01, *** indicates *p* < 0.001 (the same applies below).

The results presented in Table 2 indicate that the correlation coefficients between the 20 items included in the scale and the total score range from 0.6 to 0.9, with corresponding significance levels all less than 0.001, demonstrating highly significant statistical implications. This suggests that all 20 items have passed the homogeneity test and should be retained.

### 3.3 Reliability analysis

The reliability assessment results from Table 3 indicate that the overall Cronbach’s alpha coefficient for the questionnaire is 0.956. The Cronbach’s alpha coefficients for the 20 items, when each item is omitted, are all less than 0.956. This suggests that the overall reliability of the questionnaire is very high, and removing any single item would result in a decrease in the scale’s overall reliability. Therefore, all 20 items should be retained.

**Table 3:** Results of reliability test.

Question items	Cronbach’s α after deleting the item	Overall Cronbach’s*α*
GPRS1: I have been seeking to learn new things	0.952	0.956
GPRS2: I am usually very aware of my behavior	0.953	
GPRS3: I can work outside of my comfort zone	0.953	
GPRS4: I usually persist in studying	0.953	
GPRS5: I can tolerate uncertainty on the path forward	0.952	
GPRS6: I am committed to achieving my goals	0.954	
GPRS7: I pursue activities that are valuable to me	0.954	
GPRS8: My goals provide the direction of the have a purpose	0.954	
GPRS9: I am trying to learn from bad experiences	0.954	
GPRS10: When faced with difficulties, I can overcome negative emotions	0.953	
GPRS11: Generally speaking, I will keep trying until I figure out the problem	0.954	
GPRS12: I am good at dealing with challenges	0.954	
GPRS13: I usually pick myself up when I encounter difficulties	0.954	
GPRS14: I have the ability to achieve success	0.954	
GPRS15: I am very clear about the goal I want to achieve	0.953	
GPRS16: Continuous improvement is very important to me	0.954	
GPRS17: Feedback can help me make progress	0.953	
GPRS18: I can improve my abilities through hard work	0.953	
GPRS19: I have found that most problems can be solved	0.953	
GPRS20: I can focus on the overall situation	0.953	

### 3.4 Content validity analysis

The content validity of the Chinese version of the GPRS was evaluated by an expert panel consisting of six professionals. The I-CVI ranged from 0.833 to 1.000. Specifically, 18 items received an I-CVI of 1.000, and 2 items received an I-CVI of 0.833, both exceeding the acceptable threshold of 0.78. Furthermore, the scale-level content validity index based on the average method S-CVI was calculated to be 0.983, which is well above the recommended standard of 0.90. These results demonstrate that the Chinese version of the GPRS possesses excellent content validity.

### 3.5 Test–retest reliability

The split-half reliability of the scale is 0.736. To assess test–retest reliability, a subsample of 50 participants completed the scale again after a two-week interval, yielding a coefficient of 0.763. Independent t-tests and chi-square tests confirmed that there were no significant demographic differences (e.g., age, gender, education) between the retest subsample (*n* = 50) and the non-retest group (*n* = 526) (all *p* > 0.05). Furthermore, complementing the high Cronbach’s *α* (0.956), the McDonald’s *ω* for the overall scale was calculated to be 0.958, further confirming the excellent internal consistency of the instrument.

### 3.6 Validity analysis

**Figure 1:**
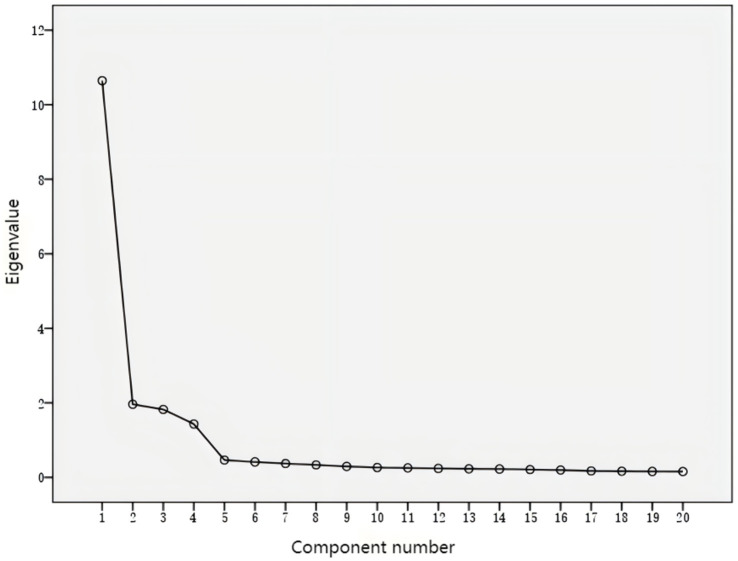
Scree plot.

As can be seen from Table 4 and Figure 1, during the factor analysis process, PAF was utilized to extract factors. Consistent with the eigenvalues greater than 1 and the scree plot, the Parallel Analysis also supported the retention of 4 factors. The results revealed a total of 4 principal components, indicating that the scale should be divided into 4 dimensions.

**Table 4:** Explanation of total variance.

Ingredients	initial eigenvalue			Extract the sum of squares and load it			Rotating sum of squares loading		
	Total points	% variance explained	Accumulate %	Total points	% variance explained	Accumulate%	Total points	% variance explained	Accumulate %
1	10.645	53.223	53.223	10.645	53.223	53.223	4.045	20.223	20.223
2	1.962	9.808	63.031	1.962	9.808	63.031	4.013	20.063	40.286
3	1.822	9.110	72.142	1.822	9.110	72.142	3.996	19.982	60.268
4	1.431	7.156	79.297	1.431	7.156	79.297	3.806	19.029	79.297
5	0.465	2.325	81.622						
6	0.414	2.068	83.690						
7	0.370	1.850	85.540						
8	0.334	1.672	87.212						
9	0.293	1.464	88.677						
10	0.263	1.315	89.992						
11	0.252	1.262	91.254						
12	0.240	1.198	92.452						
13	0.229	1.145	93.597						
14	0.223	1.117	94.714						
15	0.210	1.050	95.763						
16	0.197	0.984	96.747						
17	0.173	0.863	97.610						
18	0.165	0.825	98.435						
19	0.158	0.789	99.224						
20	0.155	0.776	100.000						

Extraction method: principal axis factoring.

### 3.7 Criterion-related validity

The correlation analysis showed that the Chinese version of the GPRS total score was significantly and positively correlated with the CD-RISC-10 score (*r* = 0.62, *p* < 0.001) (Table 5). Specifically, the four subscales—Interest, Purpose, Practice, and Hope—were also significantly correlated with the resilience score (*r* = 0.45 to 0.58, *p* < 0.01). These results indicate good convergent validity in the external sense, as the GPRS is associated with theoretically related psychological constructs.

**Table 5:** Correlations between GPRS and CD-RISC-10.

Scales/Subscales	CD-RISC-10 (r)	*p*
**GPRS total score**	**0.622**	**<0.001**
Interest	0.485	<0.001
Purpose	0.554	<0.001
Practice	0.518	<0.001
Hope	0.581	<0.001

Bold values highlight the correlation coefficient and significance level for the overall GPRS total score, distinguishing it from its individual subscales.

### 3.8 Exploratory factor analysis

The examination results indicate that the Kaiser-Meyer-Olkin (KMO) measure for the scale is 0.949, which exceeds the threshold of 0.7, suggesting that the scale is suitable for factor analysis. The Bartlett’s test of sphericity yields an approximate chi-square value of 5199.850, with a corresponding significance level less than 0.001, indicating a highly significant statistical result. This further confirms the suitability of the scale for factor analysis and suggests that the overall validity of the scale is good.

As indicated in the rotated factor matrix in Table 6, the 20 items of the scale are categorized into 4 principal components. Specifically, items GPRS1 through GPRS5 converge under the fourth principal component, which, based on the content of the items, is designated as “Interest.” Items GPRS6 through GPRS10 aggregate under the third principal component, which is named “Purpose” in accordance with the thematic content of the items; items GPRS11 through GPRS15 aggregate under the first principal component, which is named “Practice” based on the thematic content of the items. Items GPRS16 through GPRS20 converge on the second principal component, which is named “Hope” based on the thematic content of the items. All 20 items exhibit factor loadings higher than 0.5, with no instances of cross-loadings above the acceptable threshold (i.e., all cross-loadings were < 0.32), further indicating that the scale possesses strong construct validity.

**Table 6:** Rotating component matrix^a^.

Question items	Component			
	1	2	3	4
GPRS1: I have been seeking to learn new things	0.229	0.251	0.271	**0.803**
GPRS2: I am usually very aware of my behavior	0.291	0.214	0.261	**0.761**
GPRS3: I can work outside of my comfort zone	0.184	0.308	0.211	**0.784**
GPRS4: I usually persist in studying	0.246	0.207	0.241	**0.758**
GPRS5: I can tolerate uncertainty on the path forward	0.258	0.299	0.235	**0.782**
GPRS6: I am committed to achieving my goals	0.187	0.218	**0.764**	0.241
GPRS7: I pursue activities that are valuable to me	0.179	0.170	**0.812**	0.276
GPRS8: My goals provide the direction of the have a purpose	0.191	0.176	**0.826**	0.232
GPRS9: I am trying to learn from bad experiences	0.210	0.223	**0.827**	0.177
GPRS10: When faced with difficulties, I can overcome negative emotions	0.276	0.223	**0.815**	0.191
GPRS11: Generally speaking, I will keep trying until I figure out the problem	**0.801**	0.202	0.219	0.256
GPRS12: I am good at dealing with challenges	**0.819**	0.140	0.221	0.224
GPRS13: I usually pick myself up when I encounter difficulties	**0.867**	0.152	0.168	0.240
GPRS14: I have the ability to achieve success	**0.834**	0.219	0.189	0.153
GPRS15: I am very clear about the goal I want to achieve	**0.762**	0.262	0.241	0.228
GPRS16: Continuous improvement is very important to me	0.188	**0.836**	0.184	0.175
GPRS17: Feedback can help me make progress	0.185	**0.813**	0.179	0.241
GPRS18: I can improve my abilities through hard work	0.180	**0.788**	0.246	0.277
GPRS19: I have found that most problems can be solved	0.218	**0.818**	0.185	0.213
GPRS20: I can focus on the overall situation	0.178	**0.787**	0.211	0.254

Extraction method: principal axis factoring.

Rotation method: Caesar normalization maximum variance method.

^a^The rotation has converged after 5 iterations.

Bold values indicate the highest factor loading for each question item, denoting the primary component to which that item belongs.

### 3.9 Exploratory factor analysis

**Figure 2:**
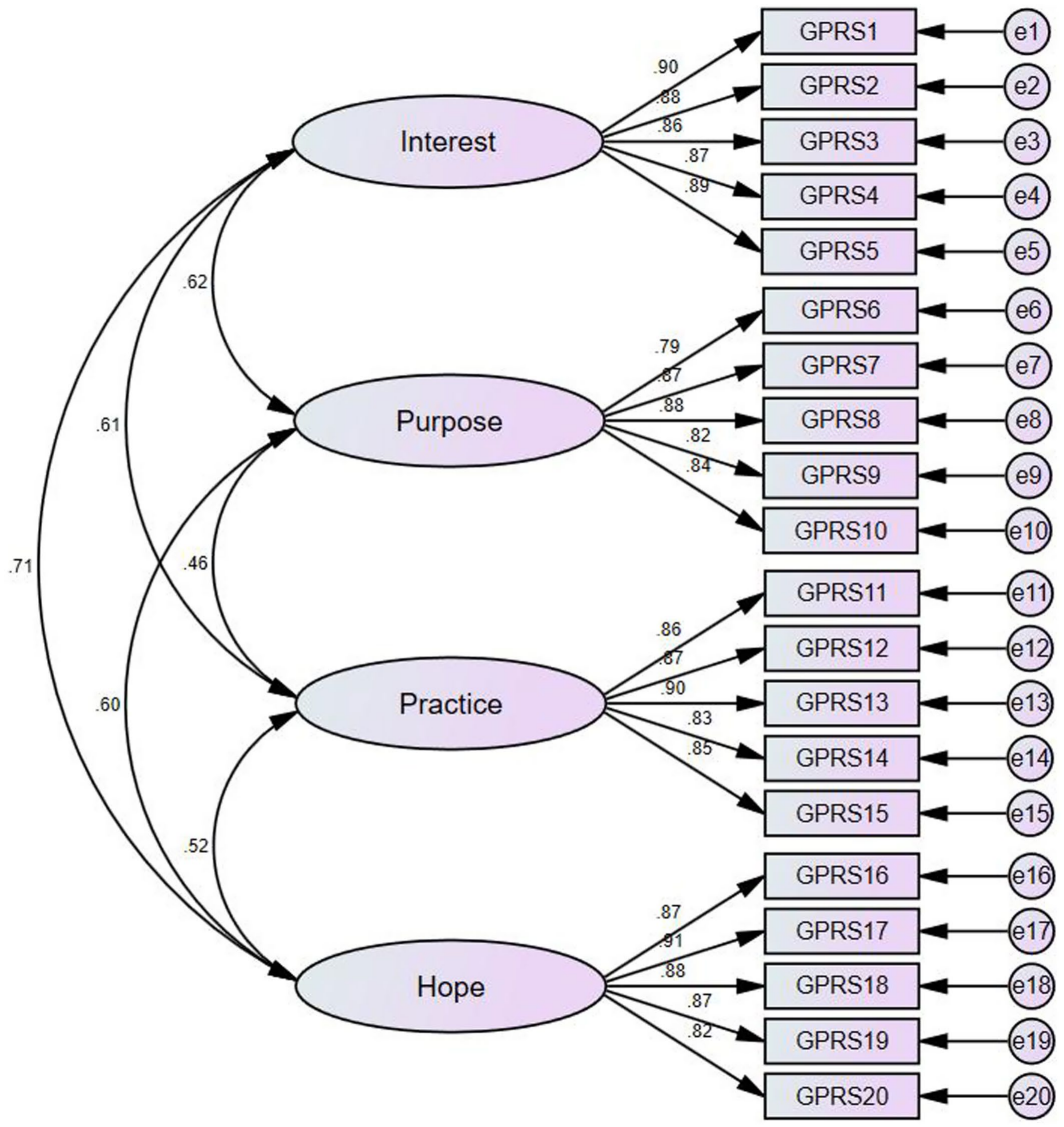
Confirmatory factor model.

In general, a chi-square to degrees of freedom ratio greater than 1 and less than 3 indicates a good model fit (Table 7). The Goodness of Fit Index (GFI) is an index of model fit, the Adjusted Goodness of Fit Index (AGFI) is an adjusted fit index, the Normed Fit Index (NFI) is a standardized fit index, the Incremental Fit Index (IFI) is an incremental fit index, and the Comparative Fit Index (CFI) is a comparative fit index. The closer the values of these indices are to 1, the better the model fit, with a general requirement of being above 0.9. The Root Mean Square Error of Approximation (RMSEA) is a measure of fit that is generally acceptable when its value is less than 0.08. A value greater than 0.1 for RMSEA is typically not acceptable. Examination of the model fit results reveals that the model’s CMIN/DF ratio is 1.473, which is greater than 1 and less than 3, indicating a good model fit. The GFI (Goodness of Fit Index), AGFI (Adjusted Goodness of Fit Index), NFI (Normed Fit Index), IFI (Incremental Fit Index), TLI (Tucker-Lewis Index), and CFI (Comparative Fit Index) all meet the criteria, reflecting a satisfactory model fit. The RMSEA (Root Mean Square Error of Approximation) is 0.04, which is less than 0.08, further suggesting a well-fitted model. The confirmatory structural equation model is depicted in Figure 2.

**Table 7:** Validation factor model fitting indicators.

Index	CMIN/DF	GFI	AGFI	NFI	IFI	TLI	CFI	RMSEA	RMR
Value	1.47	0.92	0.90	0.96	0.99	0.98	0.99	0.04	0.06

CMIN = 241.49, DF = 164, CMIN/DF = 1.47, *p* = 0.000.

### 3.10 Construct validity

Evaluate the effectiveness of the Chinese version of GPRS using combination validity, convergent validity, and discriminant validity.

#### 3.10.1 Combination reliability

The composite reliability coefficients for the four dimensions of the scale—Interest, Purpose, Practice, and Hope—are 0.945, 0.924, 0.935, and 0.939, respectively, all of which exceed the threshold of 0.9, demonstrating that the scale possesses high composite reliability.

#### 3.10.2 Convergent validity

Factor loadings and the Average Variance Extracted (AVE) are metrics for assessing the convergent validity of a scale. The minimum acceptable standard for factor loadings is 0.5, and the same threshold applies to the AVE. According to Table 8, all item factor loadings across the four dimensions exceed 0.5, and the AVE values are 0.773, 0.708, 0.742, and 0.756, respectively, all of which are above the 0.5 benchmark. Therefore, this indicates that the scale exhibits good convergent validity.

**Table 8:** Convergent validity and composite reliability of the GPRS.

Dimensions	AVE	CR
Interest	0.773	0.945
Purpose	0.708	0.924
Practice	0.742	0.935
Hope	0.756	0.939

#### 3.10.3 Discriminant validity

Discriminant validity is assessed by comparing the square roots of the AVE values of the four dimensions with the inter-variable correlation coefficients. Good discriminant validity requires that the correlation coefficients between variables are all less than the square roots of the AVE values. The square roots of the AVE values for the variables are all greater than the correlation coefficients, indicating that the overall scale has good discriminant validity.

In summary, it can be concluded that the scale demonstrates acceptable levels of overall validity, content validity, composite reliability, convergent validity, and discriminant validity.

## 4 Discussion

### 4.1 Overview and academic contribution

In recent years, the construct of grit has seen rapid development across various domains, including healthcare, education, and the workplace (Hewitt et al., 2021). Previous empirical studies, heavily influenced by Duckworth’s original conceptualization, have predominantly treated grit as a stable, trait-level personality characteristic (Chopra et al., 2025). However, this trait-based approach often limits the scope for clinical intervention. The present study addresses this critical gap by translating and validating the GPRS for the Chinese context. Unlike traditional grit scales that measure how much grit an individual has, the GPRS uniquely captures the underlying, modifiable psychological assets—Interest, Purpose, Practice, and Hope—that sustain grit (Hyun, 2025). By successfully establishing the psychometric properties of the Chinese GPRS, this study provides a crucial shift from a trait-based measurement to a resource-based framework, which is particularly vital for developing targeted psychological interventions in China’s rapidly aging population.

### 4.2 Construct validity and cross-cultural stability

A critical objective of cross-cultural adaptation is to determine whether the latent factor structure holds true in a different demographic. Through PAF and Parallel Analysis, our study confirmed a robust four-factor structure that aligns perfectly with the original GPRS development study by Schimschal et al. (2022a). This structural consistency suggests that the foundational pillars of grit psychological resources—triggering (Interest), motivating (Purpose), methodizing (Practice), and sustaining (Hope)—possess strong cross-cultural universality. Interestingly, in comparison to existing data, the total variance explained in our Chinese sample (79.297%) is notably high. Consistent with literature on adult developmental psychology, which posits that psychological resources become more integrated and crystalized as individuals age (Kurita et al., 2025), our predominantly middle-aged and older adult sample (mean age 50.53) demonstrated highly cohesive responses. Furthermore, all 20 items exhibited primary factor loadings above 0.50 with no significant cross-loadings (< 0.32), indicating that the translation maintained rigorous conceptual boundaries between the dimensions without cultural dilution.

### 4.3 Internal consistency and the redundancy trade-off

The reliability of the Chinese GPRS was exceptionally high, with a Cronbach’s *α* of 0.956 and a McDonald’s *ω* of 0.958, demonstrating excellent internal consistency and temporal stability (test–retest reliability of 0.763). However, a critical synthesis of these psychometric properties suggests a methodological trade-off that has been underexplored in previous grit literature. According to classical test theory and contemporary psychometric standards (Swan et al., 2023), α values exceeding 0.90—and particularly those approaching 0.95—often indicate item redundancy rather than merely cohesive measurement. Compared to the original English GPRS (Schimschal et al., 2022a), where linguistic nuances clearly delineate items, the Chinese adaptation reveals potential semantic overlap. For instance, within the “Purpose” dimension, items such as GPRS6 (“I am committed to achieving my goals”) and GPRS15 (“I am very clear about the goal I want to achieve”) exhibit highly correlated responses. In the Chinese linguistic context, the translation of these subtle intentional nuances often converges, leading participants to process them almost identically.

This finding is critical: while this redundancy ensures rigorous capture of the grit phenomenon and reflects the strong, unified goal-orientation of our middle-aged and older adult sample, it arguably increases respondent burden without yielding substantial additional variance. Future applications of this scale, particularly in busy clinical and public health settings, would strongly benefit from Item Response Theory (IRT) approaches (Kline and Luo, 2025) to develop a psychometrically efficient short form that optimizes the balance between comprehensive measurement and practical utility.

### 4.4 External validity and cultural nuances

To establish convergent validity in the external sense, we evaluated the GPRS against the CD-RISC-10. The moderate-to-strong positive correlation (*r* = 0.622, *p* < 0.001) confirms that grit-related psychological resources share significant conceptual variance with general psychological resilience. Crucially, however, this correlation is distinct enough (*r* < 0.85) to suggest that the two constructs are not identical.

This aligns with recent theoretical literature differentiating grit from general resilience. While resilience is frequently conceptualized as a reactive “bounce-back” capability from adversity or trauma (Terela, 2025), the psychological resources of grit—Interest, Purpose, Practice, and Hope—represent a proactive, continuous driving force for long-term goal attainment (Chen et al., 2025).

Furthermore, interpreting these four dimensions within the Chinese cultural context reveals unique theoretical nuances not fully captured in Western populations. Unlike Western individualistic paradigms where “Purpose” and “Hope” are primarily driven by self-actualization (Schuster et al., 2025), for Chinese middle-aged and older adults, these dimensions are deeply intertwined with collective family responsibilities and the Confucian ethos of Shi Gan (pragmatic action and persistence) and Le Zhi (finding intrinsic joy in the path). The robust factor loadings on the “Hope” subscale (items 16–20) underscore a resilient belief in future pathways. This culturally nuanced understanding provides healthcare and nursing professionals with a tailored framework to assess and intervene in the psychological well-being of the aging population, moving beyond Western-centric models of grit.

### 4.5 Clinical implications and limitations

The successful cross-cultural adaptation of the Chinese GPRS provides a valuable, resource-oriented tool for healthcare providers, particularly in public health and nursing contexts. Traditionally, psychological assessments in clinical settings have often been deficit-focused (e.g., screening for anxiety or depression). In contrast, the GPRS enables clinicians to adopt a strengths-based approach by identifying specific psychological assets—Interest, Purpose, Practice, and Hope. For example, in chronic disease management or geriatric care, a significantly low score in the ‘Hope’ dimension (relative to the preliminary baseline mean of 102.45 ± 15.68 observed in our cohort) could prompt nursing professionals to employ targeted meaning-centered therapies (Zhou et al., 2022) to rebuild future-oriented beliefs, rather than merely addressing physical symptoms.

Despite these clinical and theoretical contributions, several methodological limitations must be critically acknowledged. First, the cross-sectional design precludes causal inferences regarding how psychological resources influence long-term health outcomes. Second, our online survey strategy was susceptible to self-selection bias. The convenience sample was geographically concentrated (Shandong and Henan provinces) and predominantly composed of middle-aged and older adults. Consequently, the findings may not readily generalize to younger populations, rural populations, individuals with lower educational attainment, or specific clinical samples. Third, the data rely entirely on single-method self-report measures, which introduces potential common method bias and social desirability effects. Future studies should incorporate multi-informant approaches (e.g., peer or clinician ratings). Fourth, from a statistical perspective, while treating 7-point Likert scale data as continuous for ML estimation is a widely accepted psychometric convention (Sideridis et al., 2023), ordinal-specific estimators (e.g., DWLS) could further verify structural robustness. Furthermore, this initial validation lacked measurement invariance testing. Establishing demographic invariance (across age, gender, and clinical status) is a critical next step to ensure the GPRS functions equivalently across diverse subgroups. Finally, the absence of nationally representative normative data highlights the need for large-scale studies to establish definitive diagnostic thresholds for the Chinese population.

## 5 Conclusion

The Chinese version of the GPRS demonstrates satisfactory validity and reliability among Chinese middle-aged and older adults, indicating that it is a robust instrument for the assessment of psychological resources related to grit in this specific demographic, indicating that it is a robust instrument for the assessment of psychological resources related to grit. It enables healthcare providers to assess the specific psychological strengths of Chinese individuals, facilitating the transition from a deficit-based model to a resource-based model in psychological health promotion.

## Data Availability

The original contributions presented in the study are included in the article/supplementary material, further inquiries can be directed to the corresponding author.

## Footnotes

1
https://www.wjx.cn/
